# Correlation of Psychosocial Factor with Functional Outcome: One Year after Hip Fracture Surgery

**DOI:** 10.5704/MOJ.1403.013

**Published:** 2014-03

**Authors:** BU Chua, LR Bonifacio, WI Faisham

**Affiliations:** Philippine Orthopedic Center, Maria Clara St. corner Banawe Ave., Quezon City, Philippines; Philippine Orthopedic Center, Maria Clara St. corner Banawe Ave., Quezon City, Philippines; Department of Orthopaedics, School of Medical Sciences, Universiti Sains Malaysia, Kubang Kerian, Malaysia

## Abstract

**Objective:**This study investigated the correlation of 8 domains of psychosocial factors with functional outcome one year after hip fracture surgery.

A prospective cohort study of patients who had unilateral hip fracture surgery was included. A total of 89 subjects were interviewed between 3rd to 7th day after the hip surgery using the short length Multi-level Assessment Instrument and followed up after 1 year. The functional outcomes were measured through the Harris Hip Score.

All of the psychosocial factors have little to no correlation on the functional recovery of the patient but the cognitive domain had a fair correlation in Harris Hip Score (r=0.46) and is significant (p-value<0.0001)

The study did not find strong correlation between psychosocial factors and functional outcome one year after unilateral hip surgery. Although statistics have shown little effect on psychosocial factors to functional outcome, it may still be prudent to consider every aspect that may contribute to the whole wellbeing of our patients, which includes their psychosocial background.

**Key Words:**

Psychosocial Factors; Hip Fracture Outcome; Biopsychosocial; Hip Surgery; Psychosocial Background

## Introduction

Disease is traditionally defined as deviation from normal function caused by injury, pathogen and developmental abnormality. This approach however has limitations and does not cater to all the needs of the patient1. Paradigm shift into a holistic approach became more beneficial to patients as biopsychosocial model looks for potential psychological, physical, social, cognitive, affective and behavioural attributes causing the health problem. Moreover, it is important to the patient and health care provider to consider all aspects of this model to allow the patient to engage in health-promoting or treatment behaviours.

Injuries resulting from falls in older people are a major public health concern, representing the main cause of disability and death in this population. The prevalence of hip fractures among Filipino patients over 70 years and above was estimated to be 160 per 10 000. Based on this prevalence rate, it is estimated that there were about 28 000 hip fractures in 2003 and 34 000 in 2005. The numbers are expected to reach 65 000 by the year 2020 and almost 175 000 by the year 2050. In 2010, a total of 425 hip surgery was done at the Philippine Orthopaedic Center.

With the increasing magnitude of hip fracture, it is important to anticipate the necessary approach to treatment taking into consideration the factors that influence the recovery of geriatric patients with hip fracture. A study done by Mossey et.al. regarding the importance of psychosocial factor on hip fracture recovery in 219 women age 59 and older showed that poor cognitive status and post-surgical self-rated health and depression were predictive of mortality^2^. While Jelicic et.al noticed that patients with fracture experienced a great deal of pain which had a great impact on patient’s functional capacity and state of dependency. The research suggested that pain was influenced by psychological factors such as anxiety^3^. In addition, Howard et.al concluded that preoperative indicators of depression, anxiety, pain, and low self-efficacy relate directly to higher levels of pain and lower levels of function post operatively^4^.

A case control study of 387 participants by Peel et.al. suggested that psychosocial factors such as being currently married, living in present residence-, for more than 5 years, having private insurance, using proactive coping strategies and having higher level of life satisfaction have protective effect on hip fracture^5^.

A journal based on factors related to activity limitations in a group of Cuban Americans before and after hip fracture by Kirk-Sanchez showed that mental health status was related to more activity limitations prior to admission to the rehabilitation facility and early in the recovery process, but not later in the recovery process. Conversely, social support was related to activity limitations later in the recovery process, but not prior to admission or early in the recovery process^6^.

Wong et.al. studied the home readiness and recovery pattern of 50 patients after total hip replacement and found out that activities of daily living (ADLs), age and psychological status were significant factors related to home-readiness, whereas social support and hip function were significant determinants of recovery^7^.

Holmes et.al. found out that psychiatric illness was common after hip fracture and had significant effects on recovery^8^.

However, identifying psychosocial factors became difficult because of the numerous confounding factors present. Hence, with the development of different assessment tools like the Multilevel Assessment Instrument, the well-being of aged patients has been holistically considered. The tool helps clinicians gauge behavioural competence in the domains of health, activities of daily living, cognition, time use, social interaction and in the sectors of psychological wellbeing and perceived environmental stability. The present study investigated the psychosocial factors measured with short length Multilevel Assessment Instrument on 8 domains and correlated it with the functional outcome measured by the Harris Hip Score.

## Materials and Methods

### 

This is a prospective cohort study of 111 patients who had unilateral hip fracture surgery in our institution from May 2010 to April 2011. The study included patients aged 40 years and above, who, prior to sustaining a fracture, were able to walk at least 10 meters without assistance. Subjects with movement disorders such as Parkinson’s disease, Huntington chorea, hemibalismus, spastic hemiplegia or quadriplegia and those with pathologic fractures were excluded.

Prior to surgery, consent was obtained from the subjects. Between the 3rd to 7th day after the surgery, an initial structured interview using the short length multilevel assessment tool was carried out by the investigator. The responses from the subjects were written out verbatim in some questions or recorded in codes beside the questions. After the interview, the codes were transferred to the hand tabulation worksheet for standard scoring procedure. The worksheet was available for scoring indices using a summing method. In the worksheet, the answer codes were copied in a sub-index from the questionnaire to the box in the “code” column and then recorded in the re-code column. The sub-index values were computed and entered in the double-lined box provided, while the domain index scores were entered in the thick black box and values recoded were summed up.

After the surgery, the subjects were advised a standard rehabilitation program. On the 12th month of follow up, Harris Hip scores were measured and recorded by another orthopaedic resident. The records were also reviewed for possible complications that could affect the functional outcome.

STUDY VARIABLES: COGNITION: reflects subject's mental process to recall recent and remote memories.

MOBILITY: evaluates the frequency the subject was able to go out of his/her household or neighborhood and means of transportation.

ACTIVITIES OF DAILY LIVING: activities in which subject could do on his own or with assistance.

PHYSICAL HEALTH: subject’s perception of the general condition of the body which could affect the subject’s activities in time of disability of diseased state.

TIME USE: how the subject spent his time and the degree of ego-involvement in various activities, persons or ideas.

ENVIRONEMENT: reflected subject’s evaluation of his surrounding including his household and neighbourhood.

PERSONAL ADJUSTMENT: subject’s reaction to unfavorable life events such as depression, loneliness or sadness.

SOCIAL INTERACTION: subject’s social network and its availability

HARRIS HIP SCORE: A standardized measurement of hip function after surgery

**Data Analysis** Data were entered into an access database (Microsoft Excel) then analyzed on StatsDirect version 2. Categorical (qualitative) variables were reported as frequencies and percentages and continuous (quantitative) variables as means and standard deviation. All independent variables were compared to the outcome (Hip Harris score) using student’s t-test and ANOVA. The relationship of psychosocial factors to the functional outcome (Harris Hip Score, HHS) was subjected to multiple linear regressions. P-value < 0.05 is considered significant. All tests of significance are two-tailed.

## Results

A total of 111 subjects were initially interviewed, fifteen subjects were considered drop out (five died within the study period, five lost to follow up and two sustained another injury from fall, three had different types of surgery) and were not included in the Statistical Analysis.

Table I shows the demographics of patients with hip fracture. Patients mean age was 74 years , ranging from 45 to 91 years. Most of the patient were female (87.6%), age 61 to 80 years - (73.0%) and had fracture of the femoral neck area (69.7%). Partial hip replacement (PHR) was performed on 77.5% of the patients.

Most of the patients had poor Harris Hip Score 12 months after surgery (HHS < 70).Table II shows that in terms of age, patients whose age were below or equal to 60 had a higher Harris Hip Score compared to patients aged 61-80 or above 80 years old (67.7 vs 65.0 vs 62.9) and noted to be decreasing but not significant (p-value=0.79). Male patients had a higher HHS compared to female (70.5 vs 63.8) and was significant (p-value=0.01). Femoral neck fractures had a higher functional outcome than peritrochanteric fractures (66.5 vs 60.3) and was significant (p-value=0.03). Patients who underwent PHR had a higher functional outcome compared to those who had undergone dynamic hip screw (DHS) fixation (66.7 vs 57.7) and was significant (pvalue= 0.0032).

Table III shows the correlation of each psychosocial domains to functional recovery one year after surgery using Hip Harris Score. All of the psychosocial factors had little or no correlation with the functional recovery of the patient but the cognitive domain had a fair correlation on the Harris Hip Score (r=0.46) and significant (p-value<0.0001)

## Discussion

Conventionally, some orthopaedic surgeons determine the success of a procedure by physical measurements or radiographic parameters. In spite of successful surgery, some surgeons failed to notice the good functional outcome and would still report a negative post-treatment result. Hence, most of research is directed to -discovering other contributing factors for the poor functional outcome.

Current trend in the management of patients are focused on bopsychosocial model - based on a holistic approach. Through this, the treatment is based not only on the injury itself but also on its impact on the psychosocial profile of the patient, thus opening new opportunities for the health care provider to understand the reasons for their patients’ failure as gauged by functional outcome.

The results of this study have shown little to no correlation between the psychosocial factors measured and functional outcome after hip surgery. In addition, several other observed behaviours , including culture and tradition might be the reason.

The culture including the health beliefs of Filipinos are different compared to other races. As a health care provider, one has to be aware of this cultural diversity for better understanding of the health condition of the patients in catering for a more holistic approach to them. Some such beliefs and traditions were observed in this study which might have affected the result.

Majority of the subjects had reported mild to moderate pain especially during cold seasons but up to date there was no specific evidence published regarding this local experience. Hence, the subdomain score for pain resulted in a lower overall HHS rating.

The Filipino family tends to rely on direct home care rather than external health care facilities, which was observed among the subjects. This may have both negative and positive impacts on health and recovery. In cases of low income generating families, elderly patients are left unattended at home compromising the recovery from their illness. Some family members restrict elderly patients from doing household chores or even from walking because of fear of acquiring another injury, which contribute to poor functional outcome .

The Filipino people have always valued their religious virtues especially in matters of their health and well-being13. It gave them hope and strengthened their belief that they will be healed. But it could also bring hazards to their health especially if people ignored the practical measures for maintaining and treating their welfare. The “Bahala na ang Diyos” mindset is one of the beliefs that can cause harm in one’s health;by totally relying on spiritual beliefs they forget that there are medical procedures that can help them. The spirituality of the Filipino people is not being undermined here, rather promoting the notion that medicine, and its appropriate medical doctors, can go hand-in-hand with a person’s spiritual beliefs.

As age advances, there are numerous physiological declines that may contribute to poor functional outcome of patients who have undergone surgery. Factors such as retarded wound healing and altered muscle contraction due to synaptic remodeling were among them. Hence, this may explain the inverse relationship of age to functional outcome. This observation is also evident when the type of fracture and treatment were correlated to functional outcome. The patients with peritrochanteric fractures and patients who have undergone DHS fixation were older in this study (Mean Age: peritrochanteric 76.5yrs vs femoral neck 73.4yrs and DHS 75.2yrs vs PHR 74.1yrs). Moreover, there were five dropouts due to demise during the study period and were noted to be of extreme age.

The type of surgery has also been associated with different functional outcome as the metaanalysis done by Burgers et. al. which showed that patient who had partial hip arthroplasty (PHA) compared to total hip arthroplasty (THA) had less favorable functional outcome as weighted mean of the Harris hip score was 81 points after THA versus 77 after PHA. The subdomain pain of the HHS, the rate of patients reporting mild to no pain, quality of life measured with the EQ-5D and the score of WOMAC all favoured THA14. These may explain the reason for poor HHS score of 54% of the hemiarthroplasty group.

**Table I: Demographics of Patients with Hip Fracture:**
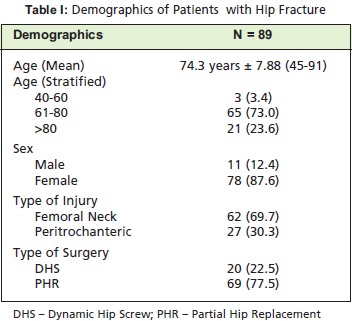


**Table II: Correlation of Psychosocial Domains with Functional Recovery 1 year after Surgery using Harris Hip Score:**
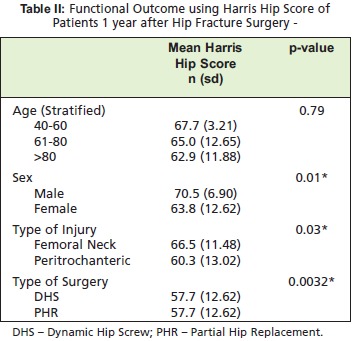


**Table III: Functional Outcome using Harris Hip Score of Patients 1 year after Hip Fracture Surgery:**
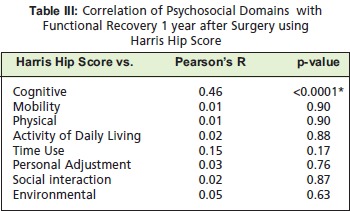


## Conclusion

This study did not show strong correlation between psychosocial factors and functional outcome one year after unilateral hip surgery. Although statistics have shown little effect of psychosocial factors on functional outcome, it may still be prudent to consider every aspect that may contribute to the whole well-being of our patients which includes their psychosocial background. Through this method, the health care provider can find a way for the improvement of his patient’s status. Therefore, assessment of patients with fracture should objectively see not only the physical or radiologic finding but also other factors that may contribute to their recovery. Thus, this study encourages every health care provider to consider the psychosocial background of his patients to maximize treatment strategies for better patient’s satisfaction. The result of this study can also be used as a reference to other researches as basis of effect of psychosocial factors on overall recovery of the patients.

The discussion and arguments on the possible effect of psychosocial factors on overall recovery and outcome, as suggested in this study, could be prudently kept in mind in the evaluation of other similar researches.
